# 

**Published:** 1892-12

**Authors:** 


VOL. XIII. DECEMBER, 1892. No. 12.

### THE International Dental Journal

A MONTHLY PERIODICAL

DEVOTED TO

### DENTAL AND ORAL SCIENCE.

JAMES TRUMAN, D.D.S., EDITOR.

JOSEPH HEAD, M.D., D.D.S., ASSISTANT EDITOR.

Publication Office,

716 FILBERT STREET, PHILADELPHIA.

PUBLISHED BY THE

INTERNATIONAL DENTAL PUBLICATION COMPANY,

51 WEST THIRTY-SEVENTH STREET, NEW YORK CITY,

—AND—

716 FILBERT STREET, PHILADELPHIA.

HARRY T. PEARCE, ADVERTISING MANAGER, P. O. BOX 151, PHILADELPHIA, PA.

Entered at the Post-Office at Philadelphia, and admitted for transmission through the mails at second-class rates.

Subscriptions must begin with the January or July number.

$2.50 per Annum, in Advance. Single Copies, 25 Cents.

PRINTED BY J. B. LIPPINCOTT COMPANY, PHILADELPHIA.

